# Epidemiological and cost analysis of burn injuries admitted to the emergency department of a tertiary burn center

**DOI:** 10.1186/s40064-016-3107-3

**Published:** 2016-08-24

**Authors:** Tolga Eser, Cemil Kavalci, Cem Aydogan, Afsin Emre Kayipmaz

**Affiliations:** 1 Department of Emergency, Faculty of Medicine, Baskent University, Fevzi Cakmak Street No: 45 Bahcelievler, Cankaya, Ankara, Turkey; 2 Department of General Surgery, Faculty of Medicine, Baskent University, Ankara, Turkey

**Keywords:** Burns, Cost analysis, Emergency treatment, Epidemiology

## Abstract

**Background:**

Burn injury is an emergency medical condition that rapidly develops as a result of tissue exposure to electrical, chemical or thermal energy. Therefore, its treatment usually begins at the emergency department. In this study we aimed to perform an epidemiological analysis of burn injuries presenting to the emergency department of a tertiary burn center, and factors affecting the cost of their medical care.

**Methods:**

Patients who presented to Baskent University Ankara Hospital Adult Emergency Department with burn injuries between January 2012 and December 2014 were studied for age, sex, time of admission, type of burn injury, clinical prognosis, mortality rate, percent burn area, and total cost of care. A total of 264 patients were enrolled. Chi square test was used for the comparison of categorical variables. Non-parametric tests were used for the comparison of continuous variables.

**Results:**

This study included 179 (67.8 %) women and 85 (32.2 %) men. The most common types of burn injuries were hot water burns and scalding. Eleven point seven percent of the patients sustained burn injuries in occupational accidents. 95.1 % of the patients were discharged from the emergency and 4.5 % of them were hospitalized. Only 1 (0.4 %) patient died. There was no significant difference between patient outcomes (discharge vs. hospital admission) with respect to the cost of care (*p* = 0.846) No significant difference was found between the cost of care of surgical and non-surgical management of burn injuries (*p* = 0.206). No significant difference was found between the costs of care of different types of burn injuries (*p* = 0.053). There was a significant difference between burn degrees with respect to the cost of care (*p* = 0.038). A significant difference was found between the costs of care of patients with a percent burn area of less than 10 % and those with a percent burn area of more than 10 % (*p* < 0.001), indicating that as percent burn area increased, a proportional increase occurred in the cost of care.

**Conclusions:**

Burn degree and percent burn area were the main determinants of the cost of care of burn injuries. In conclusion, burn injuries are preventable by taking occupational measures and raising public awareness about domestic accidents.

## Background

Burn injury is an emergency medical condition that rapidly develops as a result of tissue exposure to electrical, chemical or thermal energy. Therefore, its treatment usually begins at the emergency department. Since a significant proportion of patients affected by burn injury are admitted to the emergency department immediately after the incident, healthcare staff working in the emergency department should have a good command of these cases (Alharbi et al. 2012). After providing first medical aid at the emergency department, it is imperative that multiple departments, particularly general surgery and plastic surgery, cooperate for the management of burn injuries (Ilhan et al. 2012).

A major proportion of patients with burn injuries are discharged from the emergency department once outpatient treatment and follow-up is scheduled (Yolcu et al. 2013). When hospital admissions are taken into account, however, burn injuries are a significant source of mortality and morbidity (Sever et al. 2011). Furthermore, demanding medical care of these patients gives rise to the high cost of care depending on burn percentage, degree, as well as the duration of hospital stay (Hazar et al. 2013). Hence, taking preventive measures to avoid these injuries is the key to success for their management. Emergency physicians who assume a critical role in the management of burn injuries should necessarily have knowledge of the most common types of burn injuries and their cost of care in the emergency department.

In this study we aimed to perform an epidemiological analysis of burn injuries presenting to the emergency department of a tertiary burn center, and factors affecting the cost of their medical care.

## Methods

This retrospective descriptive study was conducted after being approved by Baskent University Faculty of Medicine Ethics Committee (Project No: KA14/282 Date of Approval: 15.10.2014). Patients who presented to Baskent University Ankara Hospital Adult Emergency Department with burn injuries between January 2012 and December 2014 were studied for age, sex, time of admission, type of burn injury, clinical prognosis, mortality rate, percent burn area, and total cost of care. A total of 264 patients were enrolled.

Hospital automation system was used to access patients’ medical data including age; sex; hour, month, and year of admission; cause and type of burn injury; occupational and suicidal nature of burn injury; burn degree; percent burn area; surgical intervention if performed; indications for admission according to the rule of nines; post-treatment clinical status, regular attendance to follow-up visits; and cost of care denominated in Euro (€). The sum for treatment cost has been obtained by adding up the invoice amounts of the inpatients and all the check-ins to the emergency department and burns outpatient department.

International Classification of Diseases (ICD) diagnostic codes T30, T31, and T32 were used to determine and record burn injuries. Statistical analyses were done with “SPSS 17.0 for Windows” software package. Chi-square test was used for the comparison of categorical variables. The distribution of continuous variables was assessed with the Kolmogorov–Smirnov test. Non-parametric tests were used for the comparison of continuous variables.

The correlation between various variables and cost of care was analyzed with the Spearman’s correlation test. A *p* value of less than 0.05 was considered statistically significant.

## Results

This study included 179 (67.8 %) women and 85 (32.2 %) men. Thirty-four (12.9 %) patients were admitted to emergency department between 00:00 and 06:00; 15 (5.7 %) between 06:00 and 12:00; 74 (28 %) between 12:00 and 18:00; and 141 (53.4 %) between 18:00 and 24:00. The monthly distribution of the emergency department admissions for burn injuries was shown in Table 1. Accordingly, patients suffering from burns check into emergency department most frequently between 18:00 and 24:00 and in June.

**Table 1:** Monthly distribution of admissions for burn injuries

Month	Number (*n*)	Percentage (%)
January	24	9.1
February	25	9.5
March	23	8.7
April	19	7.2
May	28	10.6
June	38	14.4
July	32	12.1
August	20	7.6
September	14	5.3
October	13	4.9
November	18	6.8
December	10	3.8
Total	264	100.0

Seventy-seven (29.2 %) patients were admitted in 2012; 126 (47.7 %) in 2013; and 61 (23.1 %) in 2014. One hundred and ninety-five (73.9 %) patients were admitted for hot water burns-scalding; 25 (9.5 %) for flame burns; 24 (9.1 %) for hot contact burns; 18 (6.8 %) for chemical and 2 (0.8 %) for electric burns. The most common types of burn injuries were hot water burns and scalding. Thirty-one (11.7 %) patients sustained burn injuries in occupational accidents and 233 (88.3 %) in non-occupational accidents. There was no suicidal burn injury in our study. Two hundred and fifty-one (95.1 %) patients were discharged from the emergency department and 12 (4.5 %) were hospitalized. Only 1 (0.4 %) patient died. 70.8 % of the patients involved (*n* = 187) are found to have attended to outpatient clinic controls, and 28.8 % have not.

There was no significant difference between patient outcomes (discharge vs. hospital admission) with respect to the cost of care (*p* = 0.846) (Table 2).

**Table 2:** Comparison of cost of care by patient outcome

Outcome	Number of patients	Median (€)	IQR	*p* value
Discharge	251	25.14	21.84	0.846*
Hospital admission	12	26.10	31.58	

*IQR* inter-quartile range

* Mann**–**Whitney-*U* test

It was found that there was no statistically significant difference between the gender-based groups (*p* = 0.079) (Table 3).

**Table 3:** Comparison of gender-based groups in terms of costs

Gender	Number of patients	Median (€)	IQR	*p* value
Female	179	24.22	19.26	0.079*
Male	85	27.77	28.96	

*IQR* inter-quartile range

* Mann**–**Whitney-*U* test

It was found that there was no statistically significant difference between the patients who were older versus younger than 50 (*p* = 0.593) (Table 4).

**Table 4:** Comparison of age-based groups in terms of costs

Age	Number of patients	Median (€)	IQR	*p* value
<50	214	25.25	20.71	0.593
≥50	50	26.16	26.99	

There was a significant difference between the years of admission with respect to the cost of care (*p* < 0.001). Based on the annual analysis, the highest cost of care was incurred in 2012 and the lowest in 2014 (Table 5).

**Table 5:** Comparison of the cost of burn care in different years of admission

Year of admission	Number of patients	Median (€)	IQR	*p* value
2012	77	30.29	27.50	<0.001*
2013	126	26.24	20.44	
2014	61	17.50	10.49	

*IQR* inter-quartile range

* Kruskal–Wallis test

No significant difference was found between the cost of care of surgical and non-surgical management of burn injuries (*p* = 0.206) (Table 6).

**Table 6:** Comparison of costs of care of surgical versus non-surgical management of burn injuries

Surgical intervention	Number of patients	Median (€)	IQR	Min	Max	*p* value
Not performed	258	25.09	21.61	5.96	190.93	0.206*
Performed	6	48.29	254.23	9.60	475.52	

*IQR* inter-quartile range, *min* minimum, *max* maximum

* Mann**–**Whitney-*U* test

No significant difference was found between the costs of care of different types of burn injuries (*p* = 0.053) (Table 7).

**Table 7:** Comparison of the costs of care of different types of burn injury

Cause of burn injury	Number of patients	Median (€)	IQR	*p* value
Hot water-scalding	195	25.96	21.27	0.053*
Flame	25	29.05	73.05	
Contact with hot	24	18.37	13.43	
Chemical	18	18.60	24.92	
Electric	2	23.74	–	

*IQR* inter-quartile range

* Kruskal–Wallis test

There was a significant difference between burn degrees with respect to the cost of care (*p* = 0.038). The second degree burns incurred the highest cost of care while the third degree burns led to the lowest cost of care (Table 8).

**Table 8:** Comparison of treatment costs by burn degree

Burn degree	Number of patients	Median (€)	IQR	*p* value
1. Degree	49	23.29	16.21	0.038*
2. Degree	208	26.24	24.28	
3. Degree	7	14.70	4.63	

* Kruskal–Wallis test

*IQR* inter-quartile range

A significant difference was found between the costs of care of patients with a percent burn area of less than 10 % and those with a percent burn area of more than 10 % (*p* = 0.001), indicating that as percent burn area increased, a proportional increase occurred in the cost of care (Table 9).

**Table 9:** Comparison of cost of care by percent burn area

Percent burn area	Number of patients	Median (€)	IQR	*p* value
Percent burn area < %10	250	24.93	20.17	<0.001*
Percent burn area ≥ %10	14	75.00	75.54	

*IQR* inter-quartile range

* Mann**–**Whitney-*U* test

There was a significant positive correlation between age and treatment cost (*r* = 0.184, *p* = 0.003), and also between percent burn area and treatment cost (*r* = 0.804, *p* < 0.001).

According to the descriptive statistics table, it was found that those who have 2.degree burns belong to the highest age group and those who have 3.degree burns belong to the lowest age group (Table 10).

**Table 10:** Age of the patients versus burn degree

	Median (year)	IQR
1. Degree	29.00	19.00
2. Degree	35.00	23.00
3. Degree	30.00	11.00

According to the descriptive statistics table, the most frequent reason of burns for the older age group is flame burnt while the most frequent reason for young age groups is contacted with hot objects (Table 11).

**Table 11:** Patient age versus reason of burns

Cause of burn injury	Median (year)	IQR
Hot water-scalding	32.00	22.00
Flame	37.00	21.00
Contact with hot	26.50	25.00
Chemical	31.00	13.00
Electric	31.50	–

Of the patients, those who were applied surgical intervention were older than those who were not applied surgical intervention (Table 12).

**Table 12:** Patient age versus surgical intervention

Surgical intervention	Median (year)	IQR
Not performed	32.50	21.00
Performed	38.00	38.00

Hospitalized patients are of higher age group while those who were discharged from hospital were the lowest (Table 13).

**Table 13:** Age of patient versus clinical outcome

	Median (year)	IQR
Discharge	32.00	21.00
Hospital admission	37.00	28.00

## Discussion

According to our study, there is no statistically significant difference between the patients’ discharged from the hospital versus hospitalized statuses in terms of costs (*p* = 0.846). We consider that the reason for above concern is that the cost analysis figures were including the costs arising from patients’ subsequent applications to burns outpatient department. Accordingly, we think that outpatient treatment versus inpatient treatment does not make a difference in terms of treatment costs. And despite the apparently higher costs of the patients who were applied surgical intervention; our statistical analysis has shown that there was no significant difference between the two groups (*p* = 0.206). Considering the variables such as the need for general anesthesia, the materials used, the necessity of multidisciplinary approach we expect the cost of the patients who are subject to surgical intervention, be higher. As a result of our study, we observed no significant difference between the cost versus the reason of the burns (*p* = 0.053). However, it is observed that the burns which occur due to flame exposure result in remarkably higher costs. No statistically significant data has been observed between patient age versus cost (*p* = 0.593). There is a statistically significant difference between the degree of the burn versus cost (respectively *p* < 0.001 and *p* = 0.038). This means that the cost is determined by the burnt area and its degree rather than the patient’s age.

Our study revealed that, among burn survivors admitted to our emergency department, female burn survivors were 2.1 times more common than male burn survivors. This was in contrast to a study by Saritas et al. (2011), which reported that 65 % of burn survivors who were admitted to the emergency department were male. We believe that more female survivors were admitted in our series due to the proximity of our hospital to residential areas and small-sized workplaces and its remoteness to industrial areas.

The median age of our study population was 33 years. Kowal-Vern et al. (2014) similarly reported a median age of 30 years for burn injury survivors. We think that the reason for young adults suffering from burns is observed more frequently is because these individuals are more active at home and at work. Young adults are more open to traumas and therefore the burns due to their motor-vehicle use, alcohol consumption and that they can work under heavy-duty labor requirements.

The most frequent time period for admissions was between 18:00 and 00:00 (54.4 %). DeKoning et al. (2009) also reported that admissions for burn injuries most commonly took place between 18:00 and 19:00. We considered that spending more time in low-safety, uncontrolled places and at kitchen during this period caused an increase in the burn injuries.

In our study, the most common types of burn injury were, in descending order of frequency, hot water burns-scalding, flame burns, and burns by contact with hot substances/surfaces. This finding was in agreement with that reported by Kowal-Vern et al. (2014). However, Avsarogullari et al. (2003) and Ahn and Maitz (2012) reported that flame burns were the most common types of burn injury followed by scalding. The study of Avsarogullari et al. (2003), as it can be seen in our study, consists of burn patients who check into the emergency department. As for Ahn and Maitz’s (2012) study, it did not consist of emergency service patients but inpatients of the burns unit. This can be one of the authentic properties of our study among other studies within the literature. Our finding may be a reflection of our hospital’s proximity to residential areas and small-sized workplaces.

As the majority of our cases were minor burn injuries, the median percent burn area was only 2 %. In the literature, percent burn area has been variably reported (Kowal-Vern et al. 2014; Avsarogullari et al. 2003; Aksoy et al. 2014; Sahin et al. 2011). This suggests that in our patients burn injuries may have occurred in different ways at home, workplace, or many other different settings, and thus the range of burn degrees may have been diverse.

Our study demonstrated that second-degree burns were the most common burn degree (78.78 %), a finding that was similarly reported by many other studies (DeKoning et al. 2009; Avsarogullari et al. 2003; Burton et al. 2009). Although it may show variability by different causes of burn injury, it is possible that the second-degree burn injuries were more common in patients with flame or scalding burn injuries.

According to our results, 11.7 % of burn injuries occurred in occupational accidents. Karami Matin et al. (2012) reported that occupational burn injuries constituted 28.5 % of their cases. This finding suggests that recent national measures taken for occupational health and safety have raised workers’ awareness about burn injuries and transformed workplaces to safer environments.

In our study we found that surgical treatment was used in 2.3 % patients with burn injuries. This low number possibly resulted from the mildness of burn injuries in our patients.

Similar to what Sahin et al. (2011) reported, we found that cost of care was not significantly different between various causes of burn injuries. We determined that flame burns were associated with higher cost of care, as Karami Matin et al. (2012) reported.

The cost of care of burn injuries with a percent burn area of less than 10 % was lower than that of burn injuries occupying more than 10 % of body surface area. This suggests that cost of care is affected by percent burn area. Ahn and Maitz (2012) also demonstrated that an increase in percent burn area was the primary factor leading to a proportional increase in cost of care. In patients with a greater percent burn area, hospitalization may be prolonged and complications may develop. Furthermore, increased use of workforce and medical materials lead to increased cost of care (Ahn and Maitz 2012).

We found a significant difference between burn degrees with regard to the cost of care. Burn degree and depth are similarly known to affect patient prognosis. In our study, the cost of care of second-degree burns was greater than third degree burns. Follow-up appointments of second-degree burn injuries are recommended to be spaced more closely to avoid complications. This probably led to a higher cost by increasing the number of procedures performed and medical equipment used at each visit, increasing overall cost in our study. We failed to detect any significant difference between the cost of care of patients managed surgically versus non-surgically (*p* = 0.206). Jansen et al. (2012), however, reported that early surgery allowed cost saving by decreasing the duration of hospital stay.

## Conclusions

The proximity to our hospital to residential areas and small-sized workplaces led to a greater percentage of hot water-scalding injuries that presented to our emergency department. Burn degree and percent burn area were the main determinants of the cost of care of burn injuries. In conclusion, burn injuries are preventable by taking occupational measures and raising public awareness about domestic accidents.
